# Analysis of the twelve cases of immune-related myocarditis caused by cadonilimab

**DOI:** 10.3389/fonc.2025.1671973

**Published:** 2025-10-17

**Authors:** Po Chen, Zijing Zhao, Haiying Zheng, Jinlan Li

**Affiliations:** 1 Department of Gastroenterology and Urology, The Affiliated Cancer Hospital of Xiangya School of Medicine, Central South University/Hunan Cancer Hospital, Changsha, Hunan, China; 2 Department of Pharmacy, Gansu Provincial Maternity and Child-care Hospital (Gansu Provincial Central Hospital), Lanzhou, Gansu, China; 3 Department of Pharmacy, Hunan Cancer Hospital/The Affiliated Cancer Hospital of Xiangya School of Medicine, Central South University, Changsha, Hunan, China

**Keywords:** cadonilimab, immune-related myocarditis, cytotoxic T-lymphocyte-associated protein-4, immunotherapy, programmed cell death receptor-1 (PD-1)

## Abstract

**Objective:**

This study aims to analyze the clinical characteristics of immune-related myocarditis caused by cadonilimab, thereby providing a reference for its safe clinical application.

**Methods:**

A retrospective analysis was conducted on the medical records of 12 patients diagnosed with immune-related myocarditis caused by cadonilimab at our hospital between January 1st, 2021 and December 31, 2024.

**Results:**

The incidence of immune-related myocarditis caused by cadonilimab was 2.45% (95%CI:1.30%-4.20%). Among the 12 patients who developed immune-related myocarditis, the majority were female (66.7%) and aged 65 years or younger (83.33%). The median time to onset was 49 days(IQR 27.75, 78), with 10 cases occurring within 90 days following the initial administration. 7 patients were off-label use. Additionally, 7 patients were accompanied by other immune checkpoint inhibitor (ICI)-related adverse events (irAEs), such as hypothyroidism, hepatitis and infusion reactions. 9 patients received glucocorticoid treatment and all of the 12 patients showed improvement and recovery.

**Conclusion:**

The clinical use of cadonilimab is complex, necessitating individualized monitoring for immune-related myocarditis based on the patient’s condition. Furthermore, patient education is essential to ensure medication safety.

## 1 Introduction

Cadonilimab, recognized as the first bispecific antibody globally, targets the programmed cell death receptor-1 (PD-1) and cytotoxic T-lymphocyte-associated protein-4 (CTLA-4). It has received approval from the National Medical Products Administration (NMPA) in China for the treatment of patients with recurrent or metastatic cervical cancer who have experienced failure with prior platinum-based chemotherapy. Additionally, it is approved for patients with persistent, recurrent, or metastatic cervical cancer undergoing platinum-based chemotherapy, with or without bevacizumab, as first-line treatment. Furthermore, it is applicable to patients with locally advanced unresectable or metastatic gastric or gastroesophageal junction adenocarcinoma who are receiving fluorouracil and platinum-based chemotherapy as first-line treatment (1–4). Cadonilimab exerts its antitumor effects by activating a substantial number of effector T cells through an immunosuppressive response that simultaneously inhibits the PD-1 and CTLA-4 signaling pathways. Immune-related adverse events (irAEs) represent a distinct category of adverse reactions associated with immune checkpoint inhibitors (ICIs). Despite the theoretical safety profile of cadonilimab, attributed to its tumor-enrichment effect and Fc segment silencing, which suggests a reduced incidence of irAEs, vigilant monitoring of irAEs remains imperative in patients undergoing treatment with cadonilimab. This is particularly crucial for immune-related myocarditis, which is associated with a high mortality rate; studies indicate a mortality rate of 66% when two ICIs are combined (5). Given that cadonilimab has only recently been introduced to the market, there is a paucity of reports on its associated immune-related myocarditis, complicating clinical monitoring efforts. This study will retrospectively analyze 12 cases of immune-related myocarditis, in which patients treated with cadonilimab or AK104 at our hospital between January 1st, 2021 and December 31, 2024. The objective is to investigate the clinical characteristics of cadonilimab-associated immune-related myocarditis, thereby providing a reference for its safe clinical application.

## 2 Data and methods

### 2.1 Case sources

Conduct a retrospective analysis of medical records of patients treated with cadonilimab or AK104 at our hospital from January 1st, 2021 to December 31, 2024. Specifically, focus on those diagnosed with immune-related myocarditis. Collect and document the following data: ① Basic patient information, including age, gender, weight, history of treatment and comorbidities and so on; ② Details of disease diagnosis and treatment, encompassing diagnosis, treatment plan, PD-1 status and cadonilimab dosage; ③ Information on irAEs, such as the time of occurrence, clinical symptoms, myocardial enzyme and electrocardiographic change, treatment plan and outcomes. Enter all collected data into an Excel spreadsheet and perform a thorough verification to ensure accuracy.

This research has approved by the Pharmacy Administration Committee of Hunan Cancer Hospital.

### 2.2 Inclusion and exclusion criteria

Inclusion Criteria: ①Patients must have received at least one cycle of cadonilimab treatment; ②Patients must have a confirmed diagnosis of immune-related myocarditis; ③Comprehensive data on the patient’s complete blood count, liver and kidney function, electrocardiogram, myocardial enzymes and other cardiac-related examinations must be available.

Exclusion Criteria: ①Patients with pre-existing immune-related diseases; ② Patients with pre-existing heart disease; ③Patients who have received live vaccines during the course of treatment; ④Women who are pregnant or breastfeeding; ⑤ Patients for whom the causal relationship between immune-related myocarditis and cadonilimab is determined to be likely irrelevant, pending further evaluation, or unevaluable.

### 2.3 Diagnosis and evaluation criteria of immune-related myocarditis

Diagnose immune-related myocarditis following the “Recommendations for Clinical Diagnosis and Treatment of Immune Checkpoint Inhibitors-Associated Myocarditis”,for patients suspected of having immune-related myocarditis, further tests of myocardial injury markers (troponin, creatine kinase, creatine kinase-MB, myoglobin), B-type natriuretic peptide, D-dimer, electrocardiogram etc. should be completed, and diagnosis should be made in combination with the patient’s physical signs (5). Evaluate the causal relationship between immune-related myocarditis and cadonilimab in accordance with the “Guidelines for Collecting and Reporting Individual Adverse Drug Reactions” (No. 131, 2018) (6). Grade the confirmed cadonilimab-induced immune-related myocarditis based on the “2023 Chinese Society of Clinical Oncology (CSCO) Guidelines for the management of toxicities associated with immune checkpoint inhibitors” (7). Grade 1 and grade 2 are categorized as general myocarditis, and grade 3 and grade 4 are categorized as severe myocarditis.

During the treatment process, myocardial injury markers and electrocardiogram are continuously monitored to determine whether the patient has improved or recovered. Improvement is defined as a significant improvement in the patient’s symptoms but without complete disappearance, and a decrease in myocardial injury markers levels of more than 50%. And recovery is defined as the disappearance of all symptoms, with the patient’s electrocardiogram, myocardial injury markers returning to baseline levels (8).

### 2.4 Statistical analysis

The data were processed using the R software (version 4.2.2) along with MSTATA software (www.mstata.com). The results were expressed as the median (IQR), minimum and maximum for continuous variables, and frequencies and percentages for categorical variables. Group comparisons for continuous variables were performed using the Wilcoxon rank-sum test or Kruskal-Wallis test. For comparison between groups of categorical data, we used the Fisher’s exact test for expected frequencies <5; otherwise, we used the Chi-squared test. Univariate Linear regression analysis was performed to assess the association between each individual factor (Group, IV Administration Time/D, IV Methylprednisolone Dosage, Oral Administration Time/D, and PO Methylprednisolone Dosage) and outcome variable Recovery Time/D and variables with a p-value of less than 0.1 were selected for inclusion in the multivariate analysis model.

## 3 Results

### 3.1 Incidence rate

Between January 1st, 2021 and December 31, 2024, a total of 489 patients at our hospital received treatment with cadonilimab, also known as AK104. Among these patients, 12 cases of immune-related myocarditis were identified as being associated with cadonilimab, comprising 7 cases from real-world settings and 5 from clinical study contexts (AK104-202 (1), AK104-302 (2), AK104-201 (3)), and the 5 clinical patients had been unblinded and confirmed that they received cadonilimab. The incidence rate of immune-related myocarditis caused by cadonilimab was calculated to be 2.45% (95%CI:1.30% - 4.20%).

### 3.2 Basic patient information

Individual characteristics and case summaries of patients with general myocarditis and severe myocarditis are presented in **Table 1**. In this study, among the 12 patients who developed immune-related myocarditis as a result of cadonilimab treatment, 6 patients with general myocarditis and 6 with severe myocarditis, 8 were female (66.7%) and 4 were male (33.3%). The patients’ ages ranged from 42 to 72 years, and 83.3% were aged 65 years or younger. The majority of the underlying conditions were gynecological tumors (58.3%), including 5 cases of cervical cancer and 2 cases of ovarian cancer, followed by 3 cases of lung cancer and 2 cases of gastric cancer. More than half of the patients (58.3%) were either PD-1 negative or had not undergone PD-1 testing. All 12 patients received additional anti-tumor therapies, which included paclitaxel, platinum, bevacizumab, and tyrosine kinase inhibitors such as anlotinib and apatinib. No statistically significant differences were observed between the two groups across demographic, clinical, or treatment-related variables, which demonstrated comparable distributions between groups(see **Table 1**).

**Table 1:** Basic information of patients with immune-related myocarditis.

Characteristic	Total N=12	General myocarditis N=6	Severe myocarditis N=6	P value
Source, n (%)				>0.999^1^
Real World	7 (58.3%)	4 (66.7%)	3 (50.0%)	
Clinic Study	5 (41.7%)	2 (33.3%)	3 (50.0%)	
Age				0.575^2^
Median (Q1, Q3)	57 (52, 65)	57 (53, 67)	57 (51, 65)	
Min, Max	42, 72	48, 72	42, 65	
Gender, n (%)				>0.999^1^
Male	4 (33.3%)	2 (33.3%)	2 (33.3%)	
Female	8 (66.7%)	4 (66.7%)	4 (66.7%)	
Cancer type, n (%)				0.740^1^
Gastric Cancer	2 (16.7%)	1 (16.7%)	1 (16.7%)	
Cervical Cancer	5 (41.7%)	2 (33.3%)	3 (50.0%)	
Ovarian Cancer	2 (16.7%)	2 (33.3%)	0 (0.0%)	
Lung Cancer	3 (25.0%)	1 (16.7%)	2 (33.3%)	
Stage, n (%)				>0.999^1^
IIIB	1 (8.4%)	0 (0.0%)	1 (16.7%)	
IV	7 (58.3%)	4 (66.7%)	3 (50.0%)	
Relapsed	4 (33.3%)	2 (33.3%)	2 (33.3%)	
Comorbidity, n (%)				>0.999^1^
High Blood Pressure	1 (8.4%)	1 (16.7%)	0 (0.0%)	
Hyperlipidemia	1 (8.4%)	0 (0.0%)	1 (16.7%)	
Cholecystitis	1 (8.4%)	0 (0.0%)	1 (16.7%)	
No	9 (75%)	5 (83.3%)	4 (66.7%)	
Performance status, n (%)				>0.999^1^
1	7 (58.3%)	3 (50.0%)	4 (66.7%)	
0	5 (41.7%)	3 (50.0%)	2 (33.3%)	
PD-1 status, n (%)				0.610^1^
Negative	4 (33.3%)	1 (16.7%)	3 (50.0%)	
Postive	5 (41.7%)	3 (50.0%)	2 (33.3%)	
Unknow	3 (25.0%)	2 (33.3%)	1 (16.7%)	
Surgery history, n (%)				>0.999^1^
Yes	5 (41.7%)	3 (50.0%)	2 (33.3%)	
No	7 (58.3%)	3 (50.0%)	4 (66.7%)	
Radiotherapy history, n (%)				>0.999^1^
Yes	5 (41.7%)	2 (33.3%)	3 (50.0%)	
No	7 (58.3%)	4 (66.7%)	3 (50.0%)	
Smoking history, n (%)				>0.999^1^
Yes	3 (25.0%)	1 (16.7%)	2 (33.3%)	
No	9 (75.0%)	5 (83.3%)	4 (66.7%)	
Treatment history, n (%)				>0.999^1^
No	6 (50.0%)	3 (50.0%)	3 (50.0%)	
Radiotherapy+Surgery	2 (16.7%)	1 (16.7%)	1 (16.7%)	
Radiotherapy	3 (25.0%)	1 (16.7%)	2 (33.3%)	
Surgery	1 (8.4%)	1 (16.7%)	0 (0.0%)	
Treatment line, n (%)				0.318^1^
Neoadjuvant	1 (8.4%)	1 (16.7%)	0 (0.0%)	
First line	7 (58.3%)	2 (33.3%)	5 (83.3%)	
Secone line	3 (25.0%)	2 (33.3%)	1 (16.7%)	
Third line	1 (8.4%)	1 (16.7%)	0 (0.0%)	
Combination regimen, n (%)				>0.999^1^
Tyrosine Kinase Inhibitor	4 (33.3%)	2 (33.3%)	2 (33.3%)	
Paclitaxel (Albumin-Bound)/Paclitaxel±Platinum± Bevacizumab	7 (58.3%)	4 (66.7%)	3 (50.0%)	
AK109	1 (8.4%)	0 (0.0%)	1 (16.7%)	

^1^Fisher's exact test.

^2^Wilcoxon rank sum test.

### 3.3 Administration of cadonilimab

In this study, 12 patients were included, with 11 receiving dosages ranging from 500 mg to 705 mg (equivalent to 6 mg/kg to 15 mg/kg). One patient was enrolled in the high-dose cohort of the Phase IB clinical trial investigating cadonilimab in combination with anlotinib for lung cancer, receiving a dosage of 690 mg, corresponding to a dose grade of 20 mg/kg. Additionally, one case involved off-label use for the treatment of ovarian cancer, and 6 cases involved off-label dosages, including 5 patients with cervical cancer who were treated with a Q3W regimen (see **Table 2**).

**Table 2:** Administration of cadonilimab.

Medication use		Case (number)	Specific content number)	
			Real-world Studies (number)	Clinical studies (number)
Indication	Reasonable	11	Cervical Cancer(5), Gastric Cancer(1)	Ovarian Cancer(1),Lung cancer(3),Gastric Cancer(1)
	Off-label use	1	Ovarian Cancer(1)	–
Usage and dosage	Reasonable	6	10mg/kg Q3W(Gastric Cancer 1)	12mg/kg-20mg/kg Q2W(Lung cancer 3),10mg/kg Q2W(Ovarian Cancer 1),10mg/kg Q3W(Gastric Cancer 1)
	Off-label use	6	6-10mg/kg Q3W(Cervical Cancer 5),10mg/kg Q2W(Ovarian Cancer 1)	–

### 3.4 Immune-related myocarditis

In this study, 6 out of 12 patients exhibited general immune-related myocarditis, while 6 patients experienced severe immune-related myocarditis, corresponding to an overall incidence of 1.22% for general myocarditis and 1.22% for severe myocarditis. Concurrently, 7 patients developed other irAEs including ICI-induced hypothyroidism in 4 patients, ICI-induced hepatitis in 3 patients, and infusion reactions in one patient. Among these individuals, one patient experienced three different irAEs, and 6 patients experienced two irAEs (see **Table 3**).

**Table 3:** Other irAEs occurred concurrently.

Group	Case (number)	Combined with other irAEs (number)		
		Immune-related hypothyroidism	Immune-related hepatitis	Infusion reaction
General myocarditis	6	2	0	1
Severe myocarditis	6	2	3	0
Conclusion	12	4	3	1

**Table 4** presents the clinical characteristics of immune-related myocarditis. The onset of immune-related myocarditis occurred between 18 and 391 days following the initial administration of cadonilimab, with a median onset time of 49 days(IQR 27.75, 78), which didn’t differ significantly between groups (p=0.521); notably, 10 cases manifested within 90 days post-administration. In terms of myocardial enzyme, the severe myocarditis group (n=6) demonstrated significantly higher levels of troponin I (p=0.015), all the severe patients (100%) showed an increase greater than 5 times the upper limit of normal values, while only 16.7% of the patients in the general myocarditis group were within this range. However, no statistically significant differences were observed in creatine kinase(CK), creatine kinase-MB(CK-MB), B-type natriuretic peptide(BNP), myoglobin or D-dimer levels between groups. Moreover, the incidence of fatigue symptoms in the severe group (50%) was significantly higher than that in the general group (0%) (p=0.182), although it did not reach statistical significance, it presented a clear trend of clinical differences (see **Table 4**).

**Table 4:** The clinical characteristics of immune-related myocarditis.

Characteristic	General myocarditis N=6	Severe myocarditis N=6	P value
Onset time of immune-related myocarditis			0.521^2^
Median (Q1, Q3)	69 (29, 81)	38 (24, 51)	
Min, Max	18, 249	21, 391	
Myocardial enzyme change			
Creatine kinase(CK), n (%)			0.134^2^
Normal	3 (50.0%)	0 (0.0%)	
<5ULN	2 (33.3%)	2 (33.3%)	
>5ULN	1 (16.7%)	4 (66.7%)	
Troponin I (cTnI), n (%)			0.015^2^
Normal	2 (33.3%)	0 (0.0%)	
<5ULN	3 (50.0%)	0 (0.0%)	
>5ULN	1 (16.7%)	6 (100.0%)	
Creatine kinase-MB(CK-MB), (U/L), n (%)			0.080^2^
<50	5 (83.3%)	1 (16.7%)	
>50	1 (16.7%)	5 (83.3%)	
B-type natriuretic peptide(BNP)			0.455^2^
Normal	6 (100.0%)	4 (66.7%)	
BNP elevation	0 (0.0%)	2 (33.3%)	
Myoglobin			0.545^2^
Normal	1 (16.7%)	0 (0.0%)	
<5ULN	2 (33.3%)	1 (16.7%)	
>5ULN	3 (50.0%)	5 (83.3%)	
D-dimer			>0.999^2^
Normal	3 (50.0%)	3 (50.0%)	
<5ULN	2 (33.3%)	1 (16.7%)	
>5ULN	1 (16.7%)	2 (33.3%)	
Electrocardiographic/ultrasonic cardiogram abnormalities			
Tachycardia, n (%)			>0.999^3^
Yes	5 (83.3%)	5 (83.3%)	
No	1 (16.7%)	1 (16.7%)	
Premature beats, n (%)			0.242^3^
Yes	2 (33.3%)	5 (83.3%)	
No	4 (66.7%)	1 (16.7%)	
ST-T change, n (%)			0.242^3^
Yes	1 (16.7%)	4 (66.7%)	
No	5 (83.3%)	2 (33.3%)	
Atrioventricular block, n (%)			0.455^3^
Yes	0 (0.0%)	2 (33.3%)	
No	6 (100.0%)	4 (66.7%)	
Symptom			
Fatigue, n (%)			0.182^3^
Yes	0 (0.0%)	3 (50.0%)	
No	6 (100.0%)	3 (50.0%)	
Chest tightness, n (%)			>0.999^3^
Yes	2 (33.3%)	3 (50.0%)	
No	4 (66.7%)	3 (50.0%)	
Shortness of breath, n (%)			0.567^3^
Yes	2 (33.3%)	4 (66.7%)	
No	4 (66.7%)	2 (33.3%)	
Edema, n (%)			>0.999^3^
Yes	1 (16.7%)	1 (16.7%)	
No	5 (83.3%)	5 (83.3%)	

1Wilcoxon rank sum exact test.

^2^Wilcoxon rank sum test.

^3^Fisher's exact test.

The normal ranges for above indicator: CK(50-400U/L), CK-MB(0-25U/L), cTnI(0-14pg/ml), BNP(0-900pg/ ml), Myoglobin(0-110ng/ml/), D-dimer(0-0.55ug/ml).

ULN, upper limit of normal.

Regarding electrocardiographic findings, severe myocarditis patients exhibited higher proportions of premature beats (83.3% vs. 33.3%) and ST-T changes (66.7% vs. 16.7%), though these differences did not reach statistical significance. The prevalence of tachycardia was identical between groups (83.3%), while atrioventricular block was only present in the severe group (33.3% vs. 0%). Symptom analysis revealed fatigue exclusively in severe cases (50% vs. 0%), whereas other symptoms including chest tightness, shortness of breath, and edema showed no significant differences between two groups (see **Table 4**).

### 3.5 Treatments and outcome

This study involving 12 patients, 3 patients classified as general myocarditis were monitored following the cessation of cadonilimab and did not receive glucocorticoid treatment, who improve within 1 month and recover within 70 days after the diagnosis of immune-related myocarditis. The last 9 individuals received intravenous methylprednisolone and oral methylprednisolone treatment successively, and recover within 60 days after the treatment, and the initial methylprednisolone dosage were all within the range of 1-4mg/kg. The dosage reduction plan is determined by the doctor based on the patient’s condition and the total dose of methylprednisolone used. It involves transitioning from intravenous administration to oral administration and finally to complete discontinuation. All patients complete the dosage reduction within 4 to 6 weeks (see **Table 5**).

**Table 5:** Treatment and outcome of immune-related myocarditis.

Case	Group	Initial methylprednisolone dosage (mg/kg)	IV administration time/D	IV Methylprednisolone dosage (mg)	Oral administration time/D	PO methylprednisolone dosage(mg)	Improvement time/D	Rcovery time/D
1	General Myocarditis	0	0	0	0	0	35	70
2	General Myocarditis	3.6	10	1320	30	465	10	46
3	General Myocarditis	0	0	0	0	0	21	55
4	Severe Myocarditis	2.8	27	3024	35	735	7	10
5	Severe Myocarditis	2.2	20	1800	30	550	10	15
6	General Myocarditis	3.4	18	1920	25	525	6	10
7	Severe Myocarditis	2	12	1172	40	1075	12	12
8	General Myocarditis	0.83	3	120	12	195	7	12
9	Severe Myocarditis	3	18	1285	18	300	45	60
10	General Myocarditis	0	0	0	0	0	21	45
11	Severe Myocarditis	2	8	1180	42	1365	7	7
12	Severe Myocarditis	1.35	9	1000	30	800	10	30

The univariate regression analysis revealed statistically significant associations between the IV methylprednisolone dosage (β=-0.01; 95% CI, -0.03 to 0.00; P=0.064), oral administration time/D (β=-1.05; 95% CI, -1.69 to -0.41; P=0.009), oral methylprednisolone dosage (β=-0.04; 95% CI, -0.06 to -0.01; P=0.012) and recovery time/D. However, the multivariate regression analysis revealed the IV methylprednisolone dosage (β=0.00; 95% CI, -0.03 to 0.02; P=0.666), oral administration time/D (β=-0.15; 95% CI, -3.24 to 2.95; P=0.929), oral methylprednisolone dosage (β=-0.03; 95% CI, -0.11 to 0.06; P=0.581) were not the significant predictors of recovery time (see **Tables 6**, **7**).

**Table 6:** Univariate and multivariate analysis of influencing factors (linear regression).

Characteristic	Univariable				Multivariable			
	N	Beta	95% CI	P-value	N	Beta	95% CI	P-value
Group								
General Myocarditis	6	—	—					
Severe Myocarditis	6	-17.33	-42.37, 7.70	0.205				
IV Administration Time/D	12	-1.24	-2.64, 0.17	0.115				
IV Methylprednisolone Dosage	12	-0.01	-0.03, 0.00	0.064	12	0.00	-0.03, 0.02	0.666
Oral Administration Time/D	12	-1.05	-1.69, -0.41	0.009	12	-0.15	-3.24, 2.95	0.929
PO Methylprednisolone Dosage	12	-0.04	-0.06, -0.01	0.012	12	-0.03	-0.11, 0.06	0.581

CI, Confidence Interval.

R²=0.527; Adjusted R²=0.349; Sigma=18.5; Statistic=2.97; p-value=0.10; df=3; Log-likelihood=-49.6; AIC=109; BIC=112; Deviance=2,744; Residual df=8; No. Obs.=12.

**Table 7:** Frost plot of influencing factors.

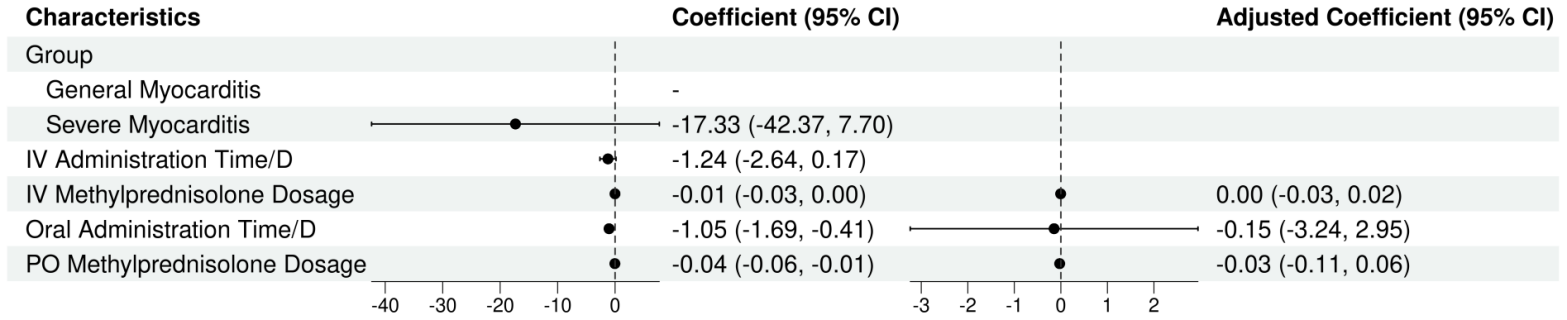

## 4 Discussion

### 4.1 Administration of cadonilimab

Currently, Cadonilimab has received approval for use in patients with recurrent or metastatic cervical cancer who have not responded to previous platinum-based chemotherapy. It is also approved for patients with persistent, recurrent, or metastatic cervical cancer undergoing first-line treatment with platinum-based chemotherapy, with or without bevacizumab, at a dosage of 6 mg/kg. Additionally, Cadonilimab is approved for patients with locally advanced unresectable or metastatic gastric or gastroesophageal junction adenocarcinoma receiving first-line treatment with fluorouracil and platinum-based chemotherapy, administered at 10 mg/kg every three weeks (Q3W). The Phase II COMPASSION-3 study (3) reported an objective response rate (ORR) of 32.3% (32 out of 99 patients) in the cervical cancer cohort treated with cadonilimab, which served as the basis for its conditional marketing approval. Subsequent findings from the COMPASSION-16 study (9)indicated that progression-free survival (PFS) was significantly greater in the group receiving the combination with cadonilimab compared to the group receiving platinum-based therapy±bevacizumab (12.7 months vs. 8.1 months, p < 0.0001), thereby further confirming the efficacy of cadonilimab in patients with cervical cancer. Similarly, the COMPASSION-15 study (2) demonstrated that among patients with advanced gastric or gastroesophageal junction adenocarcinoma, the combination of cadonilimab and the XELOX regimen resulted in a higher overall survival (OS) than the chemotherapy group (14.1 months vs. 11.1 months, p < 0.001). Additionally, OS benefits were observed in both populations with a PD-L1 combined positive score (CPS) ≥5 and CPS <5 (15.3 months vs. 10.9 months, HR 0.58; 13.7 months vs. 11.4 months, HR 0.75). In this study, out of 12 patients, 5 individuals with advanced metastatic cervical cancer received first-line treatment for recurrence. The specific regimens included 3 patients receiving a combination of bevacizumab, paclitaxel and platinum, one receiving a combination of albumin-bound paclitaxel and bevacizumab, and one patient receiving albumin-bound paclitaxel at a dosage of 6–10 mg/kg on a Q3W schedule. This dosage was an off-label use, approved by the Pharmacy Administration Committee of Hunan Cancer Hospital. Regarding the gastric cancer cohort, one patient had advanced moderately differentiated gastric adenocarcinoma with liver metastasis and positive PD-1 expression and received third-line treatment in combination with apatinib. Another patient had advanced gastric adenocarcinoma with bilateral adnexal metastasis and was enrolled in the AK104+AK109 clinical trial, with an unknown PD-1 status, receiving a dosage of 10 mg/kg on a Q3W schedule.

Recent clinical trials have demonstrated the efficacy of cadonilimab in the treatment of lung and ovarian cancers. In a study conducted by Bolin Chen et al. (10), cadonilimab administered at a dosage of 10 mg/kg Q3W was found to be effective in patients with non-small cell lung cancer, yielding an objective response rate (ORR) of 60%. Additionally, at the 56th Annual Meeting of the American Society of Gynecological Oncology, researchers (11)reported that the combination of cadonilimab with neoadjuvant chemotherapy exhibited significant anti-tumor activity and sustained clinical response in patients with advanced ovarian cancer, achieving a median progression-free survival (PFS) of 17.5 months and an ORR of 97%. Despite these promising clinical trial outcomes, cadonilimab has not yet received approval for use in the treatment of lung or ovarian cancer. In this study, 3 patients with advanced metastatic lung cancer were enrolled as clinical study participants. Among them, one patient had lung adenocarcinoma, and 2 had lung squamous cell carcinoma. These patients received anlotinib as a first-line treatment, with dosages ranging from 12 mg/kg to 20 mg/kg Q3W. Additionally, there were 2 cases of advanced metastatic ovarian cancer. One patient received bevacizumab as a second-line treatment for recurrence, which was an off-label use, while the other participated in a clinical trial involving AK104 in combination with paclitaxel and carboplatin. These findings suggest that many patients with immune-related myocarditis currently receiving cadonilimab at our hospital are utilizing the drug beyond its approved indications and dosages. The complexity of medication regimens and the limited data on the clinical use of cadonilimab pose challenges for clinical monitoring. This underscores the need for individualized monitoring of patients undergoing cadonilimab treatment.

### 4.2 Clinical characteristics of immune-related myocarditis associated with cadonilimab

In this study, among the 489 patients treated with cadonilimab, the incidence of cadonilimab-associated immune-related myocarditis was 2.45%(95CI: 1.30%-4.20%). This finding aligns with existing literature, which reports an incidence rate of 2.4% for immune-related myocarditis when PD-1 inhibitors are used in combination with CTLA-4 inhibitors (5).

To date, there have been no reported differences in immune-related myocarditis concerning gender, age or similar demographic factors. This study corroborates these findings. Of the 12 patients analyzed, the majority were female, and a higher incidence was observed in individuals under the age of 65. This demographic trend may be attributed to the study population, which predominantly consisted of patients with gynecological tumors. Notably, clinical trial cases constituted nearly half of the sample, and the median ages of diagnosis for cervical cancer (50 years) (12), gastric cancer (63 years) (13), and ovarian cancer (63 years) (14)were all below 65 years.

Within this cohort, the interval from the initial administration to the onset of immune-related myocarditis ranged from 18 to 391 days, with a median onset time of 49 days(IQR 27.75, 78). Notably, 10 cases manifested within 90 days. the findings align with the reported onset times of cadonilimab-associated immune-related myocarditis in recent case reports. For instance, Ding Di (15)described a patient with a malignant right lung tumor and cholangiocarcinoma who experienced grade 3 immune-related myocarditis after 55 days of cadonilimab treatment. Similarly, Zhang Shuo (16)reported a gastric cancer patient who developed grade 1–2 immune-related myocarditis 41 days after initiating first-line treatment with cadonilimab (625 mg) combined with the XELOX regimen. However, This study presents a deviation from the median onset time of existing literature which including more types of ICIs, such as CTLA-4 inhibitors (ipilimumab and tremelimumab), PD-1 inhibitors (nivolumab and pembrolizumab), and PD-L1 inhibitors (avelumab)and so on. Javid J Moslehi et al. (17) reported 101 cases of immune-related myocarditis, with a median onset time of 27 days (range: 5–155 days), and 76% occurred within 6 weeks after the first cycle. Syed S Mahmood et al. (18) analyzed 35 patients who developed immune-related myocarditis after using ICIs, and the results showed that the median onset time was 34 days (IQR: 21–75 days), and 81% of the cases occurred within 3 months after treatment. Tigran Makunts et al. (19)evaluated immune-related myocarditis cases based on the FAERS databases, with a median onset time of 38 days (range: 4–557 days). Charles Dolladille et al. (20) based on the analysis of the VigiBase database, showed that the median onset time of immune-related myocarditis was 28 days (range: 16–65 days); in addition, Douglas B Johnson et al. (21) reported two cases of immune-related myocarditis after the combination treatment of nivolumab and ipilimumab, with onset times of 12 days and 15 days respectively; the subsequent analysis of the Bristol-Myers Squibb safety database indicated that the median onset time of immune-related myocarditis in this combination regimen was 17 days (range: 13 - 64). The median onset time of immune-related myocarditis reported in the above studies was 17–38 days, while the immune-related myocarditis caused by cadonilimab showed a tendency to occur later, which might be caused by the different types of ICIs included in the studies. Cadonilimab was the first PD-1 and CTLA-4 bispecific antibody inhibitor, and its unique molecular structure,Fc segment silencing, and the tumor-enrichment effect made it less prone to irAEs which might to some extent demonstrate the potential advantages of the cadonilimab compared to the CTLA-4 and PD-1 combination treatment regimen. In addition, the difference in the included population is also an important cause of bias. Besides real-world patients, this study also includes clinical study patients who received more standardized treatment, more precise diagnosis, and more frequent monitoring. This will also lead to bias in the results of this study. However, it should be noted that the conclusion of this study is based on a small sample of 12 patients, and the representativeness is limited. The relevant inferences still need to be further verified by larger-scale studies.And these observations also underscore the necessity for vigilant monitoring during the initial three months of cadonilimab administration.

Among the predictive factors for immune-related myocarditis, troponin has been confirmed by multiple studies as the most reliable biomarker with early warning value. Troponin I is not only one of the strongest predictors of severe myocarditis and mortality, but regular monitoring also helps reduce disease severity and enables early intervention (22). Analysis of 748 patients further demonstrated that troponin significantly predicts adverse cardiac events (23). Moreover, elevated troponin levels were observed in 94% of immune-related cardiotoxicity cases, with both peak and final levels closely associated with poor outcomes (24). Troponin effectively predicts 30-day mortality following immune-related myocarditis (25), and a retrospective study of 90 patients further indicated that troponin is also a key predictor of one-year survival following immune-related myocarditis, with troponin levels below 1000 ng/L suggesting better long-term survival (26, 27). Dynamic monitoring revealed that troponin and N-terminal pro B-type natriuretic peptide (NT-proBNP) peak around one month after ICI initiation, decline by two months, and gradually rise again from 3–4 months onward, suggesting that both biomarkers reflect acute and chronic myocardial injury (28). Non-survivors had significantly higher levels of troponin, CK and CK-MB at presentation compared to survivors, though no significant difference was observed in BNP (29). However, some studies have also shown that elevated BNP and NT-proBNP levels are associated with severe myocarditis (30, 31). In the present study, troponin also was significantly higher in the severe immune-relted myocarditis group compared to the general group. While endomyocardial biopsy remains the gold standard for diagnosing immune-realted myocarditis, its invasive nature and procedural risks discourage its use as an initial diagnostic tool. In comparison, regular monitoring of troponin provides a convenient method for screening immune-realted myocarditis. Nevertheless, since troponin is non-specific marker, clinical diagnosis should be based on a comprehensive assessment incorporating multiple clinical examination results.

In patients with severe immune-related myocarditis, electrocardiogram abnormalities are highly prevalent, among which conduction abnormalities are strongly associated with both disease severity and prognosis. Multiple studies have reported that new-onset atrioventricular block and right bundle branch block are the most common conduction disturbances, occurring in up to 58.24% and 36% of severe immune-related myocarditis, respectively (22, 32). The presence of conduction block has been linked not only to disease progression toward more severe forms of myocarditis but also to higher mortality and worse clinical outcomes. Some studies indicate that cardiovascular mortality is significantly elevated in patients with immune-related myocarditis accompanied by conduction abnormalities compared to those without (80% vs. 16%; P=0.003) (22, 30, 33). Beyond conduction abnormalities, sinus tachycardia and prolonged QTc interval have also been identified as independent predictors of severe immune-related myocarditis (31). In this study, all cases presenting with atrioventricular block were patients with severe myocarditis, which further underscoring the critical role of electrocardiogram abnormalities, particularly conduction abnormalities, in risk stratification and prognostic evaluation of this condition.

Fatigue, chest tightness, shortness of breath, and edema are common clinical symptom of immune-related myocarditis. In the present study, no significant differences were observed in the prevalence of these signs between the two groups. Although no studies have reported that these symptoms possess predictive value for the condition, these findings underscore the importance of educating patients to recognize and report symptoms potentially associated with immune-mediated myocarditis. The guideline (5)highlights that unexplained occurrences of palpitations and chest pain should raise suspicion for immune-related myocarditis. Therefore, effective patient education in clinical settings is essential to empower patients to actively monitor their symptoms.

Meanwhile, the guideline (5) indicates that irAEs such as myositis, myasthenia gravis, abnormal liver function and abnormal thyroid function may be indicative of immune-related myocarditis. In this study, 7 of the 12 cases exhibited concurrent irAEs, suggesting that in patients treated with cadonilimab, it is imperative to consider the potential interrelationships among various irAEs rather than focusing on a single type. This approach is essential for the early identification of immune-related myocarditis.

According to the guideline (5), the first step of the management of immune-related myocarditis is suspending ICIs treatment. For patients with general immune-related myocardial injury, it is recommended to monitor myocardial enzyme levels. If these levels decrease or increase by no more than 50%, continued monitoring is advised until baseline levels are restored. If the increase exceed 50%, immediate initiation of methylprednisolone therapy(1-4mg/kg/d for 3–5 days) is warranted, for patients with severe immune-related myocardial, administer methylprednisolone at a dose of 500-1000mg/d for 3–5 days, and with the potential addition of immunosuppressive therapy for patients who do not respond adequately to glucocorticoids. In this study, all 12 patients diagnosed with immune-related myocarditis underwent drug withdrawal. 9 patients received the dosage of methylprednisolone aligning with the guideline recommendations, and respond well to methylprednisolone, no one need to start the immunosuppressive therapy. However, there is controversy regarding the selection of glucocorticoid dosage between China and European guidelines. The European Society for Medical Oncology (ESMO) (34)suggests that if myocarditis is suspected, a high-dose shock therapy of methylprednisolone at 500–1000 mg/d should be administered immediately, for 3–5 days. Some studies also indicate that: ① an initial higher glucocorticoid level (501–1000 mg/day) is significantly associated with a lower rate of sustained elevation and residual levels of troponin (18); ② and the initial dosage is negatively correlated with the incidence of major adverse cardiac events (MACE): the high-dose group (501–1000 mg/d) had an MACE event rate of only 22.0%, which was much lower than that of the medium (60–500 mg/d) and low-dose groups(<60mg/d) (P<0.001) (18, 35); ③ high-dose methylprednisolone (such as 1g/day intravenous injection) can more quickly reduce myocardial injury markers and reduce rebound. Therefore, administration of high-dose glucocorticoid for treatment optimizes clinical management and enhances patient prognosis (36). And in the present study, the recovery time in the 3 patients who didn’t receive the treatment are longer than the 9 patients received the methylprednisolone, which perhaps also means the use of glucocorticoid could reduce the recovery time. However, the negative impact of early use of glucocorticoid on ICIs efficacy also requires extra attention (35). Chen L. et al. (37)reported a case where the patient initially received the low dose (40 mg/d) glucocorticoid, but the condition progressed during the dose reduction process. Only after switching to a higher dose (80 mg/d) were the symptoms controlled. And in this study, all 9 patients who received the low or medium dose (1-4mg/kg/d) methylprednisolone treatment have recovered completely without any recurrence of the condition, and have achieved excellent therapeutic results.Therefore, although Chen L’s study further indicates that giving a full initial dose of glucocorticoid can simplify the treatment process and achieve results more quickly, whether it is necessary to use the high dosage of 500–1000 mg/d recommended in ESMO still needs to be considered.

## 5 Summary

This study conducted a retrospective analysis of the clinical characteristics of patients who developed immune-related myocarditis following the administration of cadonilimab from January 1st, 2021, to December 31, 2024. The findings indicate the following: ① The clinical application of cadonilimab in patients with immune-related myocarditis is complex, with numerous instances of off-label use. Consequently, individualized monitoring should be implemented based on the specific diagnostic and therapeutic contexts of each patient in clinical practice. ② In our small sample, no statistically significant associations were found between the development of immune-related myocarditis and gender, age, or concomitant therapy, which, however, may be due to the limited sample size. It is imperative to closely monitor the abnormal myocardial enzyme levels, such as troponin, CK, CK-MB, electrocardiographic, and related symptoms within three months post-medication. Additionally, vigilance is required for other irAEs that may signal the onset of immune-related myocarditis. Adequate glucocorticoids intervention is recommended as the preferred treatment approach. ③ There is a need to enhance patient education regarding medication, improve active monitoring and reporting capabilities, and thereby reducing the incidence of immune-related myocarditis, ensuring the safety of patients undergoing treatment.

However, this study possesses several limitations: ① This study includes both clinical study patients and real-world study patients. The strict inclusion and exclusion criteria, highly standardized intervention measures, and comprehensive and unified monitoring and recording standards in clinical study all lead to differences in the incidence and onset time of immune-related myocarditis among clinical study patients and those in the real world; ② The small sample size limits the representativeness of the clinical features obtained, thereby reducing their applicability in guiding clinical practice. In future research, as the population using cadonilimab expands, we aim to incorporate a larger cohort to achieve more representative and practical results that can effectively guide clinical use.

## Data Availability

The data analyzed in this study is subject to the following licenses/restrictions: There are ethical, legal, or privacy-related concerns with sharing the data. Requests to access these datasets should be directed to lijinlan@hnca.org.cn.
