# Correction: Analysis of the twelve cases of immune-related myocarditis caused by cadonilimab

**DOI:** 10.3389/fonc.2025.1730005

**Published:** 2025-11-06

**Authors:** Frontiers Production Office

**Affiliations:** Frontiers Media SA, Lausanne, Switzerland

**Keywords:** cadonilimab, immune-related myocarditis, cytotoxic T-lymphocyte-associated protein-4, immunotherapy, programmed cell death receptor-1 (PD-1)

Author “Haiyang Zheng” was erroneously assigned to affiliation “Department of Gastroenterology and Urology, The Affiliated Cancer Hospital of Xiangya School of Medicine, Central South University/Hunan Cancer Hospital, Changsha, Hunan, China”. The correct affiliation for “Haiying Zheng” is “Department of Pharmacy, Gansu Provincial Maternity and Child-care Hospital (Gansu Provincial Central Hospital), Lanzhou, Gansu, China”.

The original version of this article has been updated.

